# MicroRNA Signatures as Biomarkers and Therapeutic Target for CNS Embryonal Tumors: The Pros and the Cons

**DOI:** 10.3390/ijms151121554

**Published:** 2014-11-24

**Authors:** Tarek Shalaby, Giulio Fiaschetti, Martin Baumgartner, Michael A. Grotzer

**Affiliations:** Department of Oncology, University Children’s Hospital of Zurich, Steinwiesstrasse 75, Zurich 8032, Switzerland; E-Mails: tarek.shalaby@kispi.uzh (T.S.); giulio.fiaschetti@kispi.uzh.ch (G.F.); martin.baumgartner@kispi.uzh.ch (M.B.)

**Keywords:** central nervous system (CNS) embryonal tumors, medulloblastoma, atypical teratoid/rhabdoid tumors, microRNAs (miRNAs), biomarkers

## Abstract

Embryonal tumors of the central nervous system represent a heterogeneous group of childhood cancers with an unknown pathogenesis; diagnosis, on the basis of histological appearance alone, is controversial and patients’ response to therapy is difficult to predict. They encompass medulloblastoma, atypical teratoid/rhabdoid tumors and a group of primitive neuroectodermal tumors. All are aggressive tumors with the tendency to disseminate throughout the central nervous system. The large amount of genomic and molecular data generated over the last 5–10 years encourages optimism that new molecular targets will soon improve outcomes. Recent neurobiological studies have uncovered the key role of microRNAs (miRNAs) in embryonal tumors biology and their potential use as biomarkers is increasingly being recognized and investigated. However the successful use of microRNAs as reliable biomarkers for the detection and management of pediatric brain tumors represents a substantial challenge. This review debates the importance of miRNAs in the biology of central nervous systemembryonal tumors focusing on medulloblastoma and atypical teratoid/rhabdoid tumors and highlights the advantages as well as the limitations of their prospective application as biomarkers and candidates for molecular therapeutic targets.

## 1. Introduction

Embryonal tumors of the central nervous system (CNS) are biologically heterogeneous neoplasias that share the tendency to disseminate throughout the nervous system via cerebrospinal fluid (CSF) [1]. They include childhood medulloblastoma (MB), the most frequent malignant brain tumors in children, and atypical teratoid/rhabdoid tumors (AT/RT), a highly malignant group of tumor predominantly manifesting in the CNS of young children [1,2]. Survival rates of pediatric brain tumor patients have significantly improved over the last years due to the developments in diagnostic techniques, neurosurgery, chemotherapy, radiotherapy, and supportive care [3]. However, brain tumors are still an important cause of cancer-related deaths in children. Diagnosis of brain tumors is currently based on the detection of symptoms and neuro-imaging abnormalities, which appear at relatively late stages in the pathogenesis. However, the underlying molecular responses to genetic and environmental insults begin much earlier and microRNA (miRNA) networks are critically involved in these cellular regulatory mechanisms [4]. Profiling miRNA expression patterns could thus facilitate pre-symptomatic disease detection [5]. By peering into the black box of how pediatric brain tumors develop, preclinical studies, using tumor profiling and several primary and permanent cancer cell lines, have implicated many miRNAs in the development, progression and metastasis of embryonal tumors [4,6,7]. It is encouraging to note that a great deal of progress has been made recently in dissecting miRNA pathways associated with the biology of embryonal brain tumors and a number of miRNAs have been identified as potential candidates for molecular therapeutic targets [8]. Although therapeutical targeting of miRNAs has not yet been applied in clinical trials as single treatment agent regiments, it has recently been demonstrated both *in vitro* and *in vivo* that miRNA-targeted therapy may be useful in combination with conventional chemo-radiotherapy to sensitize cancer cells [9]. This review describes the current understanding of the roles of miRNAs in pediatric MB and AT/RT brain tumors, and highlights the advantages and the limitations of miRNAs as potential markers and therapeutic targets for MB and AT/RT.

## 2. miRNAs

miRNAs constitute an evolutionarily conserved class of small non-coding RNAs that post-transcriptionally suppress gene expression via sequence-specific interactions with the 3'-UTRs of mRNA targets [10]. The function of a miRNA is defined by the genes it targets and the effects exploited on its expression. A given miRNA can target several hundreds genes, and around 60% of mRNAs have predicted binding sites for one or multiple miRNAs in their UTR. Two major silencing mechanisms have been identified for miRNAs: miRNAs can inhibit translation by inhibiting translation initiation/elongation or can promote mRNA degradation. Under normal conditions, miRNAs act as moderate regulators fine-tuning gene expression, but under conditions of stress or disease, they appear to exert more pronounced functions. One of the most interesting aspects of miRNA biology is that one single miRNA can regulate multiple genes that are involved in a specific signaling cascade or cellular mechanism, making miRNAs potent biological regulators. The frequent aberrant expression and functional implication of miRNAs in human cancers, including pediatric nervous system tumors [4], and the availability of highly sensitive expression measurements techniques, have lifted these small cellular components to the ranks of ideal measurable tumor biomarkers and preferred drug targets [11]. However translation of these markers to clinical settings remains a considerable challenge and has proved more difficult than might have been expected.

### 2.1. miRNA Detection Methods: Advantages and Concerns

**Figure 1:**
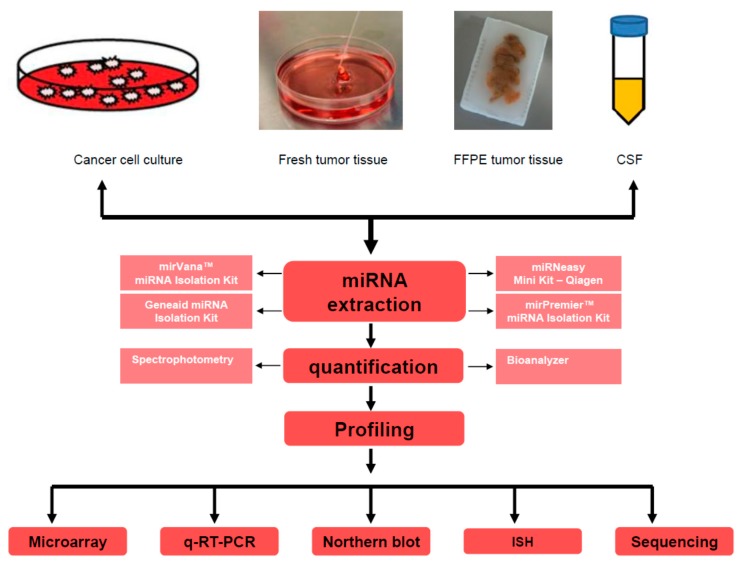
miRNA detection/profiling methods. miRNAs are usually extracted from various specimen types such as cell culture, fresh tumor tissues, formalin-fixed paraffin embedded tumors or cerebrospinal fluid. miRNA isolation methods/kits which are widely available commercially typically use a chemical extraction combined with a purification step that involves binding and eluting from a silica column. Various methods can be used to assess miRNA quality after extraction including spectrophotometry, automated capillary electrophoresis with Bioanalyzer. For cerebrospinal fluid (CSF), where usually RNA yields are too low, determining the recovery of spiked-in synthetic miRNA oligonucleotides is useful. miRNA profiling can be determined with one of the listed methods: Microarray, quantitative reverse transcription PCR (q-RT-PCR), *In Situ* Hybridization (ISH), Northern blot or RNA sequencing. FFPE: Formalin-Fixed Paraffin-Embedded.

Alterations in the expression of miRNAs in diseases can be revealed by technologies that accurately assess changes in the content of miRNAs. The development of methods for detecting miRNAs has become a research field in its own right [12]. Ideal miRNA detection/profiling method should be sensitive enough to provide quantitative analysis of expression levels, reproducible, capable of processing multiple samples in parallel, and finally, easy to perform without the need for expensive reagents or equipment [13]. Currently, various applications are available to detect miRNAs (Table 1) and determine their abundance, including microarray-based [14] and PCR-based approaches [15], Northern blot analysis with radio-labeled probes [16], *in situ* hybridization [17] and high-throughput sequencing [18] (Figure 1). However, none of these methods is perfect and all have advantages and inherent limitations [12].

#### 2.1.1. miRNAs Detection by Microarray Approach

Microarray technology is based on nucleic acid hybridization between target miRNAs molecules and their corresponding complementary probes. Microarray technology is a powerful high-throughput tool capable of monitoring the expression of thousands of miRNAs at once within tens of samples processed in parallel in a single experiment. It is usually used to conduct a genome-wide analysis of miRNA expression of normal and/or disease samples, including cancer, and to distinguish expression signatures associated with diagnosis, prognosis and therapeutic interventions [19]. The technique involves oligonucleotide probes with the same sequence as the target miRNAs which are immobilized on glass slides forming a ready-to-use miRNA microarray. The isolated miRNAs are converted to cDNA by reverse transcription, labeled with fluorescent dye and then hybridized on the microarray. After a series of washing steps to remove any unbound cDNAs, the hybridized miRNAs are detected by a microarray scanner to determine the fluorescence intensity on each probe spot, which represents the level of expression of each target miRNA from the initial RNA sample. Several technical variants of miRNA arrays have been independently developed. The main differences include probe design, immobilization chemistry, sample labeling, and microarray chip signal-detection methods [20]. These genome-wide miRNA microarray platforms have the advantage of generally being less expensive than the other profiling methods and yet they allow large numbers of parallel measurements. The main disadvantage of these techniques is that they are unable to identify novel miRNAs. Application of microarray technology is also challenged by several innate properties of miRNAs, mainly the short length of miRNAs that offers little room to fine-tune the hybridization conditions. The low abundance of miRNAs fraction of total cellular RNA (<0.01%) and the imperfect specificity for miRNAs that are closely related in sequence and may differ by a single nucleotide, represent yet additional challenges which significantly decrease the sensitivity and specificity of microarrays. Another drawback of microarray technology is the lack of ability to perform absolute quantification of miRNA abundance [21]. Nevertheless microarrays are considered to be the best tool for comparing relative abundance of specific miRNAs between two states such as “experimental” *vs.* “control” or “diseased” *vs.* “healthy” samples [21]. Finally, a large quantity of RNA is required for testing, and microarray chips are also very expensive to fabricate. However, if routinely implemented in basic and clinical research laboratories, microarray-based miRNA expression profiling has the certain potential to lead to the discovery of novel biomarkers and therapeutic targets (reviewed in [21]).

#### 2.1.2. Real-Time-PCR-Based Detection of miRNAs

Real-time PCR is the gold standard for gene expression quantification. Although global expression profiling assays are useful to provide a broad overview of the presence and the regulation of miRNAs, these data normally require a confirmation by more specific approaches. To date the most commonly used method to detect specific miRNAs is the real-time PCR analysis. This approach relies on reverse transcription of miRNA to cDNA, followed by quantitative PCR (qPCR) with real-time monitoring of reaction product accumulation. Commercially available customizable plates and microfluidic cards can be designed either to examine a small set of miRNAs or to provide more comprehensive coverage by large-scale profiling of hundreds of miRNAs [21]. Because of its high level of sensitivity, accuracy and its ability to differentiate between single base mismatches, quantitative reverse transcription PCR (q-RT-PCR) is accepted as a powerful technique in comparative expression analysis in life sciences and medicine [12]. Another appealing aspect of this approach is the practical ease of incorporation into the workflow of clinic laboratories. A hurdle in performing highly parallel q-RT-PCR is the challenge for scientists to design a conventional PCR assay from miRNAs averaging around 22 nt in length. The optimal reaction conditions may also vary substantially between miRNAs owing to sequence-specific differences in primer annealing. A limitation of q-RT-PCR technique is that the reaction cannot identify novel miRNAs. Another major drawback is the requirement for special equipment that is likely not available in most laboratories, which makes widespread application of this method difficult [22].

#### 2.1.3. Northern Blot Analysis for miRNAs Detection

Northern blotting was used to identify the very first miRNAs, and still remains a gold standard for miRNA expression analysis [23]. Although it is fairly time consuming and requires large amounts of RNA, it is the only approach that will visualize the expression product of a miRNA. After isolation of total RNA from cells or tissue, the small RNAs are fractionated by electrophoresis on a high percentage gel. After transferring these small RNAs from the gel onto a nitrocellulose membrane, to allow detection by hybridization with fluorescent or radio-labeled probes that are complementary to the target miRNA, the RNA is fixed onto the membrane by UV cross-linking and/or baking the membrane. Because of the small size and the low abundance of miRNA molecules, the use of an oligonucleotide probe with high sensitivity is essential for the detection of a given miRNA [24]. Northern blotting technique allows the validation of predicted miRNAs by the examination of their expression levels and the determination of their sizes. However, there are several technical limitations that prevent the routine use of Northern blotting as miRNA expression profiling tool in clinics. Mature miRNA molecules are very short and their abundance in total RNA is also very low, leading to a poor sensitivity of routine Northern blot analysis. Moreover this method is very time-consuming and not practical in clinical studies in which the detection of a large number of miRNAs might be required; its use in diagnostic is also limited by the relatively large amount of RNA sample required and the multiple handling steps.

#### 2.1.4. *In Situ* Hybridization (ISH) for miRNA Detection

ISH is an important tool used to detect miRNA accumulation in tissue sections or fixed cells. The technique uses labeled complementary nucleic acid sequences to detect a single strand of DNA or RNA. The advantage of ISH is the ability to monitor specific miRNA expression at the cellular or even at sub-cellular level, which results in asemi-quantitative analysis of miRNA expression. However, detection of miRNAs by ISH is technically challenging because of the small size of the target sequences and the low expression level of some miRNAs. Hence the use of this technique as a routine tool for miRNA expression profiling tool in diagnostics will likely remain limited by the low quantification power and by the low throughput nature of this methodology.

#### 2.1.5. miRNA Detection by High-Throughput Next-Generation Sequencing Platforms

All of the techniques described above depend on hybridization and are restricted to the detection and profiling of previously identified miRNA sequences. Next generation sequencing platforms do not depend on hybridization and do not require previous information. The sequence-based methods for miRNA profiling determine the nucleotide sequence of miRNAs and involve RNA isolation, ligation of linkers to both 3' and 5' ends, reverse transcription, and PCR amplification, followed by the “massively parallel” sequencing of millions of individual cDNA molecules. Bioinformatic analysis of the sequence reads identifies both known and novel miRNAs in the data sets and provides relative quantification using a digital approach [22]. High-throughput sequencing methods permit high-resolution views of expressed miRNAs over a wide dynamic range of expression levels and the discrimination of miRNA family members that differ in only a nucleotide. Unlike profiling of miRNAs based on microarray techniques, deep sequencing measures absolute abundance and allows the discovery of novel miRNAs. Therefore deep sequencing methods are emerging as the best tool for studying miRNA expression also because they can reliably be used for miRNA detection in fresh frozen paraffin-embedded specimens, thus making large-scale clinical studies possible. In addition high-throughput sequencing of miRNAs have successfully revealed the differential expression of miRNAs in several cancerogenic and pre-carcinogenic lesions [13,25], suggesting that they can be used in diagnostics for early detection and for assessment of tumor aggressiveness [12].

Potential limitations are high costs and the computational infrastructure required for data analysis and interpretation [26].

In summary, microarray technology based methods appear to be best suited for detection of miRNA profiles, although RT-PCR may be the method of choice in a clinical settings if a combination of only a few miRNAs is used as marker. In addition, if it is important to know in which cell type a specific miRNA is expressed, ISH methods may be required. Despite being expensive some consider next-generation sequencing as the method of choice for studying small RNA expression. In contrast to the other methods, this platform is hybridization-independent, more sensitive and accurate in discriminating miRNA family members that could differ by only a single nucleotide and can be used to simultaneously detect known and yet unknown miRNAs.

### 2.2. miRNA Target Determination

In the past decade the number of discovered genes encoding miRNAs has risen exponentially and researchers have gained substantial in-depth knowledge of the basic mechanism of action of miRNAs, but the main challenge still remaining is the identification of the tangible direct targets of these molecules, to understand how they regulate so many biological processes in both healthy and diseased tissue. Many technologies have been developed in the past few years, all with their own pros and cons [27]. Initial insight into miRNA targets can be obtained bioinformatically through a number of available programs that predict potential mRNA targets for individual miRNAs. Although it is important to confirm these predictions using miRNA target validation techniques, bioinformatic target prediction is often the first step toward defining the function of a specific miRNA. Currently, there are a number of freely available programs, such as miRanda (http://www.microRNA.org), microCosm (previously known as miRBase targets, http://www.mirbase.org), Targetscan (http://www.targetscan.org), or PicTar (http://pictar.mdc-berlin.de) that predict which mRNAs can potentially be targeted by a given miRNA or which miRNAs might be able to target a certain gene of interest. Main characteristics that these programs use to determine whether a miRNA can potentially target an mRNA include sequences complementarity between the 5' seed region of the miRNA (usually 2–8 bases) and the 3'-UTR sequence of a target gene. Thermal stability of the mRNA/miRNA duplex is often also taken into account; reviewed in [22]. After the identification of potential miRNA target genes, the physical binding of miRNA to candidate mRNAs and the subsequent translational modulation needs to be confirmed. A common approach for miRNA target verification is by cloning the 3'-UTR of a predicted mRNA target into a luciferase reporter. By linking the target UTR to the luciferase reporter, a change in luciferase signal will indicate whether a miRNA can bind to the UTR and regulate the expression of the gene. For this approach cell lines are chosen due to their transfectability (for example, HEK293 cells); however, tissue-specific miRNA biogenesis and binding warrants that these studies be performed in relevant cell line models resembling the tissue of origin [27].

## 3. miRNA Implications in CNS Embryonal Tumors

miRNAs are implicated in diverse biological processes including cell self-renewal and pluri-potency [28] and as etiologic genes in various human malignancies [29]. Patterns of miRNA expression have been shown to distinguish tumor types and to predict tumor behavior [30]. However, their role in malignant pediatric brain tumors remains largely unexplored.

### 3.1. miRNAs Associated with Neuronal Development and Disorders

Studying the expression pattern of miRNAs during mammalian brain development, Kim *et al.* [31] identified miR-103, miR-124a, miR-128, miR-323, miR-326, miR-329, miR-344 and miR-192-2 to be expressed in the rat cortex and cerebellum during neural differentiation and development, reviewed in [32]. Using human embryonic cells that differentiate into neurons upon retinoic acid treatment, Sempere *et al*. showed that miR-9/9, miR-103-1, miR-124a, miR-124b, miR-128, miR-135, miR-156 and miR-218 are coordinately induced during the neural differentiation process [24]. Remarkably some of these miRNAs were reported to be also active in neuro-developmental disorders [33]. The notion that cancer is fuelled by self-renewing stem cells is gaining prominence, and so is the idea that miRNA can direct cell fate. Yu *et al.* has brought the two fields together by showing that a single miRNA molecule “miRNA let-7” can regulate stem-ness in cancer cells [34]. The important role of stem cells both in normal tissue and cancer development has driven much of the research into neural cancer stem cell biology. Silber *et al*. reported that miR-124 and miR-137 induce differentiation of neural stem cells and glioblastoma stem cells [35]. Desanomi *et al*. reported that miR-34 deficiency is involved in the self-renewal and survival of cancer stem cells, and that in cancer cells lacking functional p53, restoration of miR-34 was able to re-establish the tumor suppressing signaling pathway [36]. The research in this area has recently led to the identification of specific miRNA genes responsible for embryonal stem cells (ESCs) proliferation and differentiation and for the initiation and progression of cancer stem cells [34,35,36,37,38]. miR-17/92 is an example of an miRNA associated with MB and neuronal stem cell biology. It promotes neural stem cells development by modulating its cell-fate decision and it is also involved in cancer stem cell maintenance and in MB biology [39]. Other miRNAs such as miR-7, miR-9 and repression of miR-124 were reported to be associated with MB biology and neuronal differentiation [40,41,42] while miR-125b, miR-324-5p, and miR-326 regulate SHH signaling in cerebellar granule neuron precursors and MB cells [4]. Interestingly, specific miRNAs such as miR-302 were recently found to be capable of reprogramming the cancer cells back into a pluripotent embryonic stem cell-like state, which then could be induced to mature into tissue-specific cells [43]. Together these reports suggest that miRNA expression is vital for normal as well as abnormal embryonic stem cells development that can lead to cancer stem cell initiation, reviewed in [36]. Cancer stem cells have been identified, isolated and characterized recently in embryonal neural malignancies including MB [44,45,46,47,48]. With the knowledge that MB harbors cancer-initiating cells with stem cell properties, scientists are making a great effort to understand the involvement of aberrantly expressed miRNAs in embryonal cancer stem cells and to elucidate the mechanisms which distinguish these cells from normal stem cells. Functional studies on miRNAs within the cancer stem cells of MB will be crucial for elucidating the mechanisms behind oncogenesis in these deadly malignancies and might reveal novel therapeutic targets.

### 3.2. Biological Relevance of miRNAs in MBs

MBs are primary malignant embryonal tumors of the central nervous system and represent more than one fifth of all pediatric brain tumors [1,3]. While the prognosis has traditionally been based on conventional histopathology and clinical staging, in recent years it has become apparent that the inherent biology of the tumor plays a significant part in predicting survival [49]. Recent advances in molecular biology and integrated genomics have led to an improved understanding of the genetic abnormalities and alterations in cell signaling pathways associated with MBs. Four distinct molecular subgroups of MB have been identified (WNT (wingless), SHH (sonic hedgehog), Group 3, and Group 4) [50,51,52]. Profiling of these subgroups revealed distinct genomic events, several of which represent prognostic and predictive biomarkers as well as targets for therapy [50,51,52,53,54]. Specifically, stratification of patients into their respective subgroups has profound prognostic impact, wherein therapy can be de-escalated in patients with favorable prognosis, and intensified therapy or novel agents can be considered in patients with poor prognosis [55]. However, despite considerable progress, more effort is still needed to fine-tune the identification of specific biological alterations that could be targeted by molecular specific therapies. In this scenario miRNA research is emerging together with the established evidence regarding the key roles of these molecules in this cancer. Despite the fact that miRNAs are involved in the tumorigenesis of a range of different tumors, the knowledge about the prognostic, diagnostic, and/or therapeutic target potential of these molecules in brain cancer, especially MBs, is still at an early stage [8].

#### 3.2.1. miRNAs Function as Oncogenes or Tumor Suppressors in MB

The class of genes that function as tumor suppressors and oncogenes in MB have recently been expanded to include the miRNA family [41,42,56,57] (Table 1). miR-124a was amongst the first to be characterized as a tumor suppressor miRNA in MB [58]. This miRNA is a brain-enriched miRNA found to be expressed in the external granule cells of the cerebellum, reported to be cells of origin of MBs [59,60,61]. miR-124a was found to be decreased in MB cells compared to normal cerebellum and restoration of its function inhibits MB cell proliferation [58]. Pierson *et al.* [58] reported that cyclin dependent kinase 6 (CDK6) and solute carrier family 16, member 1 (SLC16A1) are functional targets for miR-124a. Both genes were reported as an adverse prognostic marker in MB [62,63]. Ferretti *et al.* [4] identified a set of four up-regulated miRNAs (let-7g, miR-19a, miR-106b and miR-191) that are capable of distinguishing MB patient samples with aggressive *vs.* non aggressive histological MB variants. Furthermore, the authors observed in MB samples impaired expression of specific miRNAs that are known to be expressed during neuronal development such as (miR-9, miR-125a, miR-128a, miR128b and miR-181b), suggesting that some of these miRNAs might be involved in MB tumorigenesis [64]. Interestingly, these miRNAs were previously identified before in other brain tumors such as glioblastomas [65]. Experimentally, increasing the expression of miR-9 or miR-125a decreased MB cells survival and promoted MB tumor growth arrest suggesting a role for both miRNAs as tumor suppressors [66]. In a recent survey of miRNA expression in pediatric brain tumors including MB, miR-216, miR-135b, miR-217, miR-592 and miR-340 were found to be upregulated, whereas miR-92b, miR-23a, miR-27a, miR-146b and miR-22 were found to be downregulated, compared to normal brain tissue [67]. MB genome-wide miRNA expression profiling studies have revealed close associations of miRNA clusters with molecular and clinical subgroups [31,63,68,69]. In these studies miR-21 [70] was found to be up-regulated across all MB subgroups compared to normal cerebellum, while a miR-17/92 cluster was reported by Northcott and colleagues to be significantly up-regulated specifically in SHH-driven MBs [63,71]. Their results were confirmed in a report by Uziel *et al.* that showed miR-17/92 promoting proliferation of MB cell lines *in vitro* and contributing to the development of MB *in vivo* [63,71]. Other labs also associated miR-17/92 and miR-106b-25 cluster over-expression with both human and mouse SHH MBs [69,72]. Moreover miR-17/20 and miR-19a/b inhibitors decreased tumor growth in SHH MB cells *in vitro* and in animals bearing flank or cortical SHH MB allografts [73] suggesting that inhibitors targeting the miR-17/92 cluster could be therapeutically useful in SHH MBs [74]. Over-expression of other oncogenic miRNA clusters such as miR-183-96–182 were reported in MB subgroups characterized by genetic amplification of MYC, and not in SHH MBs [68] and promote metastasis, a hallmark of MYC aggressive MBs [75]. Ferretti and colleagues found additional miRNAs differentially regulated in MYC over-expressing MBs [69]. However, it is not known whether these miRNAs are directly regulated by MYC; reviewed in [74]. Cho *et al.* discovered a previously unidentified molecular subgroup, genetically characterized by gain of MYC copy number, miR-183-96–182 upregulation and significantly associated with lower rates of event-free and overall survival [76]. For the WNT MB subgroup, miR-193a, miR-224/miR-452 cluster and miR-148a were reported to have potential tumor/metastasis suppressive activity and were found to be over-expressed in WNT signalling-associated MB [77]. To summarize, knowledge about miRNA expression signatures in distinct MB subtypes provides new insight into the molecular pathogenesis of MB tumors and highlights the hope of potential translation of such valuable information into therapy.

**Table 1:** MicroRNAs (miRNAs) involved in medulloblastoma (MB) biology as oncogenes or tumor suppressors.

miRNAs (miRs)	Targets	References
**Oncomirs**		
miR-21	PDCD4	[78]
miR-517c, miR-520g	miR-517c WNT/JNK signaling, miR-520g ABCG2	[79,80]
miR-221, miR-222	p27*^Kip1^*	[6]
miR-183-96–182 cluster	Undefined, however knockdown of the full miR-183-96–182 cluster results in enrichment of genes associated with apoptosis and dysregulation of the PI3K/AKT/mTOR signaling axis	[68]
miR-17/92	TSP-1, Bmi-1, PTEN, PP2A	[63,81,82]
miR-214	Gli1	[4]
miR-30b, miR-30d	Undefined, Both miRNAs are part of an amplicon containing the KHDRBS3 gene on 8q24.22 in MB cell lines	[83]
miR-106b	p21	[69,84]
**Tumor Suppressors miRNAs**		
miR-125a	t-TrkC	[69]
miR-9	REST/NRSF, Hes1	[39,69]
miR-125b, miR-324-5p, miR-326	PKM2, SMO, Notch	[4,85]
let-7	RAS, STAT3	[69,86]
miR-199b-5p	HES1, Notch pathway, ErbB2	[87]
miR-124a	CDK6, SLC16A1, REST, BAF34a, RB1,t-TrkC	[35,58,60,88,89]
miR-218	EGFR, Bcl-2, B-catenin and MAPK9	[84,90]
miR-31, miR-153	miR-153 is found in high ErbB2 expressing MB	[69,90]
miR-128a, miR-128b, miR-181b	Bmi-1	[64,91]
let-7 miRNA family	HMGA2	[7]

#### 3.2.2. miRNAs Associated with MB Metastasis

Metastases are responsible for the majority of MB-related mortality, yet our understanding of the molecular circuitry coordinating this process is fragmentary. miRNAs are believed to play a key role as suppressors or promoters of metastasis according to their mRNA targets [92,93]. miR-21 up-regulation is associated with metastasis and cell migration in a variety of solid tumors including breast, lung, colon, prostate, pancreas and stomach cancers, as well as brain tumors such as glioblastoma [78,94,95]. Our lab recently reported the upregulation of miR-21 in MB cells, while miR-21 inhibition decreased MB cell migration *in vitro*. Subsequent studies have identified other miRNAs as either promoters or suppressors of metastasis in MBs [96,97]. miR-106b was found to be overexpressed in MBs and directly interacting with PTEN. Inhibition of miR-106b in MB cells reduced cell migration and invasion potential [96]. In another interesting study, miR-182 was found to be able to contribute to leptomeningeal metastatic dissemination in non-SHH MB [75]. Additionally miR-193a, miR-224/miR-452 cluster, and miR-148a were reported as exerting potential metastasis suppressor activity [31], while miR-199-5p was described as an inhibitor of MB metastasis [87]. Along the same lines, miR-219 suppresses invasion and metastasis through targeting CD164 [97].

### 3.3. Clinical Relevance of miRNAs in MBs

**Figure 2:**
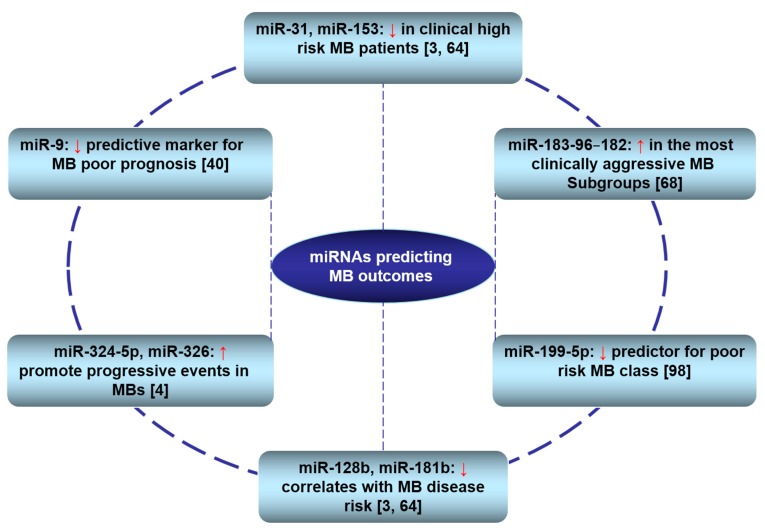
miRNAs associated with outcome prediction in MB patients. ↑: up-regulated; ↓: down-regulated.

Risk stratification and prognosis assessment have become a major concerns in the era of personalised medicine. Although gene expression profiling has reached a plateau in this regard, recent miRNA studies show new great promises [31,63,68], reviewed in [98]. Multiple reports have noted the utility of miRNAs for the prognostics and risk stratification of MBs (Figure 2). Moreover, miRNA expression profiles have been used to distinguish tumor histo-types. For example the oncogenic group miR-let7g, miR-106b, miR-191 and miR-19a are up-regulated in more aggressive MB anaplastic histotype with respect to classic and/or desmoplastic tumors [69]. The potential clinical utility of miRNAs extends beyond the realm of tumor classification to other important clinical measures, such as prognosis and treatment response. The low expression of tumor suppressor miRNAs, such as miR-199-5p, was found to be predictive for poor prognosis [87]. Low expression of miR-128b and miR-181b has been reported to correlate with MB disease risk [64,69], while both miR-31 and miR-153 are down-regulated in clinical high-risk MB patients [56,64,69]. On the other hand, oncogenic miRNAs, such as miR-125b, miR324-5p, and miR-326, promote progressive events in MBs [4]. Moreover, Weeraratne and colleagues reported that increased expression of the miR-183-96–182 cluster characterizes the most clinically aggressive subgroup and is associated with genetic amplification of MYC [68]. The same trend was observed when the miR-183-96–182 cluster was found to be significantly associated with non-SHH MBs, while miR-182 promotes metastasis, a hallmark of aggressive MBs [75]. Our group recently identified miR-9 as a methylation-silenced tumor suppressor that could be a potential candidate predictive marker for MB with poor prognosis [39]. The investigation demonstrated that LC/A MB samples possess lower miR-9 expression compared to the other variants and that miR-9 under-expression is associated with poor prognosis in 34 patient samples of MB. The lower overall survival probability of patients with low miR-9 expression that also tends to have a more severe pathological grade suggests a strong trend towards prognostic significance. Moreover, in line with previous reports, high expression of the main miR-9 target gene (*HES1*, hairy and enhancer of split 1 homolog), correlated significantly with lower overall survival in a distinct cohort of 129 MB samples [76]. In summary, although the full potential of miRNAs as prognostic factors awaits the results of larger prospective studies and further verification steps, the above mentioned data imply that in the near future miRNAs may have a wide clinical applicability as diagnostic and prognostic biomarkers for MB patients.

### 3.4. Biological Relevance of miRNAs in CNS Atypical Teratoid/Rhabdoid Tumors

CNS AT/RT are highly malignant central nervous system neoplasms that usually affect very young children and are typically deadly despite very aggressive treatment [99,100]. A standardized and effective approach for the treatment of this tumorremains elusive [101]. Rhabdoid tumors predominantly arise in the kidney and brain, but they can also be found in a deep axial location, such as the neck or the paraspinal region [102]. Different forms of rhabdoid tumors can be similar in their aggressiveness, histological features, and loss of function of INI1/hSNF5 mapping on chromosome 22 [103,104]. From clinical experience, infants and children with rhabdoid tumors respond very poorly to chemotherapy and radiotherapy [105,106,107], although this remarkable resistance to both cytostatic drugs and radiotherapy has not yet found a convincing explanation at the molecular level. Because of the rarity of the disease, to date few miRNAsin AT/RT have been studied (Table 2). Sredni *et al*. [6] demonstrated that overexpression of miR-221/222 inhibited the expression of the tumor suppressor and inhibitor of cell cycle p27*^Kip1^*. The authors suggested that deregulation of miR-221/222 expression might be one of the factors contributing to oncogenesis and progression of AT/RT through p27*^Kip1^* down-regulation and speculated that anti-miR-221/222 therapy might be an option for the treatment of these very aggressive and unresponsive tumors. Comparing miRNA expression in pediatric brain tumors and normal tissue controls, AT/RT samples showed high expression of miR-520b, miR-629, miR-221, miR-498 and miR-373, while miR-140, let-7b, miR-139, miR-153, and miR-376b were under-expressed [67]. To find new potential therapeutic targets for the treatment of AT/RT, Zhang *et al.* [7] have recently searched for novel genomic aberrations by investigating the copy number and expression alterations of let-7a3/let-7b miRNA and correlated them with expression of high-mobility group AT-hook 2 (HMGA2) oncoprotein, a target of let-7 miRNA family, in 18 AT/RT samples. Their analysis demonstrated that HMGA2 was highly over-expressed in 83.3% of AT/RT tissues while let-7a3/let-7b miRNA copy number and expression were reduced. Restoration of let-7 miRNA or knockdown of HMGA2 expression significantly suppressed proliferation and colony formation and almost abolished the invasive potential of G401 Wilms’ tumor cell line. The authors suggested that the HMGA2 oncoprotein plays a critical role in the pathogenesis of AT/RT development and reconstitution of let-7 miRNA may provide a novel therapeutic strategy for the treatment of AT/RT patients.

**Table 2:** miRNAs involved in atypical teratoid/rhabdoid tumors (AT/RT) biology as oncogenes or tumor suppressors.

miRNAs (miRs)	Targets	References
**Oncomirs**		
miR-517c, miR-520g	miR-517c WNT/JNK signaling, miR-520g ABCG2	[79,80]
miR-221, miR-222	p27*^Kip1^*	[6]
miR-520b, miR-629, miR-498, miR-373	Undefined	[67]
**Tumor Suppressors miRNAs**		
miR-140, let-7b, miR-139, miR-153, miR-376b	Undefined	[67]
let-7 miRNA family	HMGA2	[7]
miR-9	Undefined	[40,69]

## 4. miRNAs as Reliable Clinical Markers: Advantages and Challenges

With the increasing implication of miRNAs in cancer development and progression, significant efforts are underway to use miRNAs as novel biomarkers with clinical applications [108,109,110]. miRNA expression profiling has been used to characterize embryonal and differentiated tissues of the nervous system [111], to discriminate cancer from normal tissue [70,93], and to differentiate primary from metastatic brain tumors [112]. Moreover, miRNAs identify individuals with increased disease risk, thus representing potential novel prognostic factors [113]. Of note, miRNAsncan also be detected circulating in several body fluids, including plasma, serum, cerebrospinal fluid, urine, and saliva [114,115,116,117,118]. Because of the significant differences that have been reported between the circulating miRNA expression profiles of healthy individuals and those of patients, it is encouraging to state that circulating miRNAs are likely to become a novel class of non-invasive and sensitive biomarkers for diagnosis and other clinical applications in human diseases [119]. In fact the use of circulating miRNAs as potential biomarkers is a rapidly evolving study field [108,120] and the number of publications has increased rapidly over the past two years. In the context of CNS diseases, several studies have demonstrated significant presence of specific miRNAs in CSF samples in patients with CNS lymphoma, glioma [117], metastatic brain cancers [116], and Alzheimer’s disease [121]*.* However, the true source of miRNAs in the body fluids and their exact secretory mechanism is still relatively unknown [122].

### 4.1. Process of Biomarker Development

The pathological progression from pre-neoplasia to cancer is accompanied by alterations in gene sequences, expression and function. Changes that occur exclusively, or more commonly, in cancer cells can be detected by specific molecular assays in tumor biopsy or in body fluids, and used as molecular indicators of cancer. These indicators/markers are useful in detecting cancer at early stages, assessing tumor burden, monitoring disease progression, and determining response to therapy [123]. Biomarkers can be broadly classified as prognostic or predictive markers that estimate disease-related patient trajectories and predict patient-specific outcome to different treatments [124]. Different conceptual phases of biomarker development have been proposed: phase one is the preclinical exploratory step that often begins by comparing tumor tissue with non-tumor tissue in order to identify characteristics unique to tumors that might lead to tests for clinical cancer detection. After the discovery stage, biomarkers must clear a number of practical hurdles before they can be considered for clinical practice. They have to undergo multiple defined stages of assay confirmation and validation. According to the US Food and Drug Administration (FDA), a “biomarker” is a “biomarker” that is measured in an analytical test system with well-established performance characteristics and for which there is an established scientific framework or body of evidence that elucidates the physiologic, toxicologic, pharmacologic, or clinical significance of the test results (Phase one) [125]. Phase two is a crucial subsequent step that evaluates the performance of biomarkers in distinguishing normal controls from cases with tumors (clinical validation) by testing the markers in tissues retrospectively collected from research cohorts [126]. This is best performed collaboratively with clinical and epidemiology centers. Phase three is the prospective screening phase. Ideally, biomarkers should be validated analogously in prospective, well-controlled clinical studies of large samples of diverse patients across multiple institutions. Phase four is the cancer control phase, comprising large-scale population studies to evaluate both the role of the biomarkers in detecting disease and the overall impact of screening in the population [127,128]. Not all biomarkers however will need to progress consecutively through all of the phases outlined here.

### 4.2. miRNAs as Reliable Markers

The ideal biomarkers should fit a number of criteria: To show satisfactory predictability and the ability to be inspected during onset, progression and/or regression of the disease, to be easy to assay and applicable to automatic high-throughput technology [108,129,130,131]. Today, most of the biomarkers detected in cancer patients are proteins, such as prostate specific antigen (PSA) for prostate cancer, alanine aminotransferase (ALT) and aspartate aminotransferase (AST) for hepatocellular carcinoma, *etc*. However, the protein assay procedure is not always easy to apply in clinical diagnosis and remains labor-intensive. Moreover the complexity of protein composition, post translational protein modifications, proteolysis and denaturation, as well as the low abundance of the proteins of interest add to the challenge of using proteins as successful biomarkers [132]. As a result, only a few of those protein-based biomarkers have been approved to be used in the diagnosis of cancer. Considering the limitations of current cancer conventional biomarkers, the use of miRNAs as tumor markers has aroused intense research interests, in particular after the discovery that profiling of miRNA expression patterns was more useful than the equivalent mRNA profiles [133]. In addition, contrary to mRNA which is not stable in formalin-fixed paraffin-embedded tissue, miRNAs expression seems to well correlate between fresh and FFPE samples, possibly due to the small size and resistance to degradation of miRNAs [134,135]. Stable miRNAs have also been detected in body fluids including serum, plasma, urine, and other biological fluids as well as CSF [108,136]. Together these features make miRNAs extremely attractive for clinical research, since archived FFPE tissue and biological fluids is most often available, reviewed in [137].

It is estimated that miRNAs regulate around 30% of human protein-coding genes and their dysregulation has been associated with the development and progression of cancer [137]. Because miRNAs play many roles in diverse aspects of cancer, such as proliferation, apoptosis, invasion, metastasis, and angiogenesis, their expression pattern can be used to characterize tumor type, stage, or other clinical variables. They also play an active role in the etiology or progression of cancers by regulating the expression of oncogenes and tumor-suppressor genes. Thus, they could have potential for future cancer diagnosis and prognosis. Recently various studies have successfully identified the histotype of tumors of unknown origin according to miRNA expression profiles [138]. miRNA profiling has also shown promise in classifying human cancers [133], defining malignant status [139], and accurately identifing cancer tissue origin. Furthermore, evaluation of miRNA expression has been found to be of great prognostic [140,141] and diagnostic value [142,143]. Additionally, miRNA expression was shown to provide promising biomarkers for the outcome prediction of a wide array of human cancers [138,143,144], as well as for predicting the response to chemo-radiotherapy [145,146].

Utilization of automatic high-throughput technology for precise measurements is a fundamental analytical issue that needs to be fulfilled in molecular marker assays. In this regard the widespread and comprehensive use of high-throughput miRNA technologies, such as amplification-based quantitative real-time PCR (q-RT-PCR), hybridization-based microarrays, and sequencing-based next-generation sequencing (NGS) technologies, have enabled the accurate quantification of miRNA expression and the identification of miRNAs uniquely expressed in cancers [147]. Advanced technologies not only help to identify the oncogenic and tumor suppressor potentials of miRNAs, but also assist in providing a more comprehensive understanding of their underlying mechanisms and pathways. In summary, features of miRNAs such as rich information content, great discriminatory power, stability, accessibility in different specimen types, possibility of being evaluated from different sources, and finally, and importantly, their potential for highly sensitive measurement, have qualified them as reliable biomarkers. In fact, some tests using miRNA as biomarkers for clinical diagnosis are now available. Rosetta Genomics offers three different tests designed to identify specific miRNA signatures and use them to accurately diagnose diseases and predict their progress. Another company, Asuragen, has developed tests to diagnose pancreatic and colorectal cancer, as well as leukemia [12].

### 4.3. Challenges

Application of miRNA profiling approaches for many malignancies, including CNS embryonal tumors, has produced hundreds of candidate biomarkers for detection and prognostication [40,64,68,69,87], yet none have become established in clinical practice. Fundamental issues have slowed the progress of clinical deployment, mainly the lack of clinically relevant animal models for miRNA research. Therefore, the creation of a suitable, well-defined and validated animal model is essential to elucidate the role of miRNAs in embryonal tumors and to transfer knowledge from bench to bedside and so to address clinical questions [148]. Other factors contributing to the failure of candidate biomarkers to realize clinical utility include those inherently associated with conventional biomarkers such as (i) lack of collaborative research efforts; (ii) availability of tumor tissue samples; (iii) reliability and reproducibility of results; (iv) variability of biomarker assessment methods; (v) expenses involved with assessing the marker status; (vi) the need for developing new miRNA assessment technologies; and (vii) lack of cellular and clinical database resources.

#### 4.3.1. Lack of Collaborative Research Efforts

Most of the miRNA alterations in embryonal brain tumors have been identified by research groups studying small numbers of retrospectively collected tumor samples, and using different experimental methods and protocols. The generated data and results are therefore difficult to compare, statistically underpowered, and do not successfully result in the development of fully validated biomarkers [149,150,151,152,153]. Hence there is a great need to facilitate the transition and incorporation of miRNA biomarker research from single research groups to a more collaborative approach. Large strategic research partnerships and international collaborative projects should be developed between pediatric brain tumors labs. Efforts to develop and validate miRNAs as biological markers for pediatric brain tumors should be shared between different international labs, research responsibility should be divided, and duties should be clear. Initiatives are also needed tounitebasic and clinical researchers and promote their collaboration. It is vital to link clinicians and researchers of molecular measurements in order that experimentation becomes clinical reality. Collaborative and integrative research, proper communication and understanding among pediatric brain tumors labs worldwide are key methods of uniting the skills necessary for developing successful biomarkers.

#### 4.3.2. Availability of Tumor Tissue Samples

To date, most of the investigations into the use of miRNAs as biomarkers have been performed mainly in cell lines or animal models while other studies included only small cohorts of patient samples that were not validated in prospective clinical trials. A final decision for human use must be based on results derived from clinical trials with an adequate number of patient samples that represent a proof of concept for the field of miRNAs as biomarkers. The sensitivity and specificity of a molecular marker cannot be fully realized until careful testing is carried out in tumor specimens and compared with normal controls [123,154,155,156,157,158]. Therefore, access to well-preserved human tissues accompanied by high quality clinical data is a prime issue to be considered in order to examine the relationship between molecular changes and clinical variables or outcomes. The collected specimens usually include tumors, as well as adjacent normal tissues when possible and are associated with a spectrum of information that usually includes the collection time, the tissue composition and the alterations within that reflect the type and stage of disease (histopathology). The small number of available tumor tissue samples however has disappointingly limited the output of researchers in the field of rare tumors, such as embryonal brain tumors, and the clinical translation of many candidate miRNA markers is stalled by the lack of well-characterized tissue samples [154,155,156,157,158]. An analysis of biomarker publications showed that many individual studies report high association between specific biomarkers and disease outcome; however when the same marker is subsequently compared with larger studies, or meta-analyses, the effect size is often significantly smaller than initially believed [159]. This has brought recognition to the urgent need to improve the availability and access to tissue resources. Tumor collections by single institutions or individual scientists are mostly not sufficient to address these resource problems. Therefore, improved tools such as biobanks, one of the key resources in the fight against cancer, are certainly needed [160,161,162]. However, unlike other research tools such as cell lines and animal models, the biobank boom spawned an increase of regulations and guidelines, which has created controversies, particularly about the importance and definition of informed consent [163,164,165] that is required before biobank samples can be collected and used in research. Unclear, conflicting, or burdensome regulatory requirements and lack of agreement among clinicians, investigators, and regulators as well as the nature of this consent and how it is obtained add another layer of complexity to the sample collection matter. Some European guidelines take the view that general consent is acceptable to use samples for future, as yet unspecified research projects; US and Canadian policy follows a more rigorous standard of consent [163].

#### 4.3.3. Reliability and Reproducibility of Results

Acquisition of highly reproducible data is necessary to produce reliable markers that could be used in clinical situations. However, the lack of standardization that is manifested in the variability in experimental conditions, methodology, measurements, protocols, and platforms, is another very important factor that challenges the process of miRNAs biomarker validation process. There is skepticism and legitimate debate within the research community on what is needed to be done to translate laboratory findings into tangible clinical applications [166]. The skepticism is partially derived from some inflated expectations, which are frequently followed by disappointment when the original results of certain investigations could not be reproduced. As is known from the field of microarrays, data is often incomplete or incompletely annotated and the analyses are hard to reproduce [167]. For example, four different research groups have reported miRNA profiles for pancreatic cancer, with impressive discrepancies between the results [12,168,169,170].

#### 4.3.4. Variability of Biomarker Assessment Methods

Another key factor that hampers the validation of biomarkers and contributes to the failure of candidate biomarkers to realize clinical utility is the multitude and variety of sample processing, RNA extraction and expression measurement/assessment methods. Equally important are the differences in specimen type, for example FFPE *vs.* fresh frozen samples. Studying miRNA profiles involves sample collection; miRNA detection; analytical platform and protocols, data processing; statistical analysis, and clinical interpretation [12]. A variety of these analytic techniques exists, each with specific biases that can greatly influence the relative weight of certain miRNA molecules in the tested sample. No wonder there is often a low correlation of results obtained from different platforms or even from the same platform using kits and reagents from different vendors. These are especially pronounced when a highly sensitive technique like sequencing is used. Currently there are no universally implemented guidelines for the collection, preparation, and extraction of samples for miRNA analysis. Standardization of these assays is a challenge for the near future. Data normalization, an often underestimated aspect of data processing, is as well crucially important and a major concern in obtaining accurate results [171]. Therefore, it is important to be methodologically consistent across different samples and steps must be taken to carefully select miRNA-profiling platforms and data analysis methods for the acquisition of clinically meaningful and dependable data. Furthermore, standardization of miRNA biomarker evaluation is definitely needed.

#### 4.3.5. Expenses Involved with Assessing the Marker Status

It has been difficult to translate molecular markers to the clinic, owing to not only the above mentioned factors, but also due to the vast expense that is involved in developing the necessary platforms and technology for testing different assays in a prospective clinical trial. Indeed economic and business considerations can slow cancer biomarker development. Appropriate finance to provide clinical opportunities for the evaluation of new technologies, reagents and assays is lacking and industrial companies do not play a role in most pediatric disease research. Many of the miRNA diagnostic and prognostic markers that have been reported in the literature are in the public domain and lack intellectual property protection. Companies have shied away from developing clinical tests in the absence of this protection. It has to be said, however that the miRNA-based biomarker development problems are not only concentrated in the pediatric brain tumor field. There has been a huge increase in intellectual endeavors concerning research devoted to the discovery and validation of other disease biomarkers within both the biological and clinical sciences during the last decade. For instance, in searching for the term “biomarker” in the literature database, more than 660,000 published articles could be found. However, these enormous investments and academic output have not yet translated into the expected integration of new biomarkers for patient care [172].

#### 4.3.6. The Need for Developing New miRNA Assessment Technologies

There is still no clear-cut consensus as to what is the best approach to analyze large-scale miRNA profiles. It is expected that these issues will settle with time, as techniques become more robust and analysis methods stabilize [173]. Refining, and/or developing, new detection and measurement technologies with improved analytic tools that allow more detailed examinations of the molecular and cellular signatures of pediatric brain tumors is an important task that is needed for the development of successful miRNA biomarkers and efficacious target gene therapy discovery [174]. It is estimated that non-coding RNAs involved in cancer may include over 1500 miRNAs. Such complexity clearly highlights the need for ultra-high resolution technology for robust quantitative miRNA measurements and data acquisition as well as to analyze already discovered potential markers in a cost-effective fashion [123]. Development of new measurement technologies therefore is central to successful biomarker development and should be strongly encouraged.

#### 4.3.7. Lack of Cellular and Clinical Database Resources

Databases play a major role in cancer research at the cellular and clinical levels, with a central role currently played by expression array data. Global electronic database gathering and storing a wide range of data for genetic variations and miRNA expression profiles that are pediatric brain tumor-relevant must be developed and sustained [174]. Web-service technology linking molecular level databases and biobank data as well as software platforms for data and document management, facilitating remote communication between biomarker development’s concerned parties should be encouraged. A common information infrastructure for data exchange, analysis and modeling and meta-analysis methodologies should be put into practice, thereby allowing researchers the choice between going through lots of papers published over the past several decades on individual experiments, or more simply using high throughput datasets.

All in all we are just starting to uncover the huge potential of miRNAs as novel biomarkers for medicine. However, as mentioned above, conceptual and technical issues still need to be overcome. Moreover there are still many questions remaining unanswered in understanding miRNA biology and function, and as yet, not all the enzymes and proteins involved in miRNA production and processing are known [175]. We have not identified other factors contributing to miRNA-target gene recognition, other than seed sequence complementarities, nor the actual regulation of miRNAs at the transcriptional level. It is still unclear how cancer cells manipulate miRNAs and other regulators to promote their own survival and growth under stressed tumor microenvironments [176]. A more thorough understanding of miRNA biology is certainly needed before candidate biomarkers realize clinical utility. However, despite current limitations, miRNA-based biomarkers constitute an exciting field in biomedical research and it is likely to become a routine approach to generate individual patient profiles and allow targeted therapeutic intervention.

## 5. Therapeutic Potential of miRNAs

### 5.1. miRNAs as Druggable Targets

As noted above, several miRNAs are altered in pediatric brain tumors disease states when compared to the normal brain. Whether this differential expression occurs as a consequence of the pathological state or whether the disease is a direct cause of this differential expression is currently unknown. Nonetheless, since miRNAs are deregulated in cancer, it is thought that normalization of their expression could be a potential method of intervention. In this vein, several therapeutic mechanisms have been put forth and are described [177,178,179]. Generally there are two approaches to develop miRNA-based therapeutics: miRNA antagonists [70,180] and miRNA mimics/replacement [181]. miRNA antagonists inhibits endogenous miRNAs that show a gain of function in cancer tissues. This approach is conceptually similar to short interfering RNAs (siRNAs). It usually involves the introduction of anti-miR or antago-miR that bind with high affinity to the active miRNA strand and results in its degradation. On the other hand, miRNA mimics/replacement are used to restore a loss of function. This approach aims to re-introduce miRNAs into diseased cells that are normally expressed in healthy cells. miRNA mimics can be delivered systemically using technologies that are also used for therapeutic siRNAs [181].

There are several significant advantages of miRNAs for becoming a new class of drug targets. Their small size and known and conserved sequence make them attractive candidates from a development standpoint. Moreover, many genetic or oligonucleotide-based gain- and loss-of function studies have shown very pronounced phenotypes in rodents and even large animal models, whereas miRNA manipulation under baseline conditions usually does not exert overt effects. Furthermore, and importantly, the direct downstream targets of a single miRNA are commonly related genes that function in a comparable cellular process or signaling cascade. This implies that targeting of a single miRNA will probably result in a dramatic effect due to the combinatorial effect of gene expression changes in all these related downstream targets, reviewed in [182].

### 5.2. miRNAs as Predictors and Modifiers of Chemo- and Radio-Therapy

Perhaps the most promising application of miRNAs might lie in the estimation of outcome and response of well-established anti-tumor therapies, such as chemotherapy and radiation. For example, it has been recently shown that alterations in miRNA expression profiles could provide predictive information about sensitivity or resistance of certain tumor types to different treatments; alternatively, or in addition, changes in expression during a therapy might offer a tool for treatment response monitoring. Finally, modification of miRNA expression by up- or down-regulation may possibly enhance sensitivity to the applied chemo- or radio-therapy, reviewed in [183]. Extensive profiling studies on cancer cell line, animal xenographs, and tumors, have associated miRNA expression in cancer cells with chemo- and radio sensitivity, both with regards to predicting or modulating sensitivity. Several miRNAs have been found to predict sensitivity to anticancer treatment, while others were shown to influence sensitivity to chemo- or radio-therapy *in vitro* and/or *in vivo*. There are many potential roles for miRNAs in altering chemotherapeutic response that have been reported in the literature. For example, miRNA replacement or silencing may be used to augment chemotherapeutic effects [184,185] or alternatively to base the selection of traditional chemotherapies on their ability to deregulate specific panels of miRNAs [186]. Perhaps the most clinically applicable approach is to use miRNA signatures to guide chemotherapeutic decisions. *In vitro* studies have identified miRNAs whose expression patterns correlate with drug response [187,188]. However, global profiling studies have been used as classifiers of responders *vs.* non responders in various cancers [186,189]. miRNAs may modulate the DNA damage response, thus sensitizing tumor cells to both chemotherapy and radiotherapy [190,191]. The role of miRNAs as sensitizers to radiotherapy is of particular importance given the fact that many pediatric brain tumors in infants and young children require combinations of chemotherapy and high doses of spinal radiotherapy as optimal modes of treatment. In this case infants could be treated with a substantially less toxic treatment strategy.

### 5.3. Challenges of miRNA-Based Therapies

Although there are many reasons to be excited about miRNAs as a new class of drug targets, some aspects of miRNA and anti-miRNA biology are still relatively unknown and the majority of the promising miRNA targeting approaches are still at their preclinical stage. The optimization of the stability of miRNAs, the improvement in delivery systems, and targeted drug delivery as well as the understanding and control of off-target effects of miRNA therapeutics, are the main challenges for their future development. Similar to small interfering RNA therapies, difficulties of specific delivery, and the insufficient uptake for effective target inhibition challenge the development of miRNA-based therapies. Despite the fact that chemical adaptations in oligonucleotides, such as cholesterol conjugation and phosphorothioate backbone modifications, were able to overcome these obstacles, impaired biological activity and increased toxicity usually accompanied the resulted improved delivery to tissues [192]. Moreover it is expected that tumor types and context will add to the complexity and heterogeneity of response to any miRNA therapy strategies. Blood-brain barrier (BBB) forms another important hurdle for effective miRNA treatment of malignant brain cancer. Hence, effective brain delivery for miRNAs may require the design of particular therapeutic approaches that overcome this physiological obstruction, such as the use of nanoparticles, immunoliposomes, peptide vectors or carrier-mediated transport through the BBB, reviewed in [193]. New therapies targeting miRNAs or their target genes may best be applied in the future together with molecular profiling of cancers for clinical stratification and selection of combination therapies.

## 6. Concluding Remarks

miRNAs are revolutionizing the field of cancer research. The emerging role of their dysregulation in human cancer has raised exciting opportunities as well as challenges for their clinical application in the capacity of cancer detection and prediction.

In the therapeutic arena, the realization that the inappropriate production of individual miRNAs contributes to several aspects of carcinogenesis suggests that inhibition of overexpressed oncogenic miRNAs or substitution of tumor suppressive miRNAs could become a novel treatment strategy or a promising candidate for treatment response prediction or for modulation of conventional anticancer treatment sensitivity. With the advent of high-throughput technologies for the global measurement of miRNAs, research has shown miRNAs to have potential as biomarkers for the diagnosis and prognosis of pediatric brain tumors. However most miRNA studies have been limited to the preclinical discovery stage. The challenge remains to optimize and to validate miRNA biomarkers through carefully designed translational/clinical studies. Such studies require careful practical consideration of the best methods for sample collection, miRNA isolation, quantification, and data analysis and importantly to translate this wealth of discovery into clinical management of pediatric brain tumors patients. It is likely that significant progress will be achieved in the usage of miRNAs in cancer diagnosis and outcome prediction in the near future, and undoubtedly, the coming years will bring exciting new therapeutic strategies based on the targeting of miRNA in the treatment of human cancer. It is hoped that some of these will be efficient and beneficial for pediatric brain cancer patients.
